# The Role of Marketing Practices and Tobacco Control Initiatives on Smokeless Tobacco Sales, 2005–2010

**DOI:** 10.3390/ijerph16193650

**Published:** 2019-09-28

**Authors:** Mary Hrywna, Irina B. Grafova, Cristine D. Delnevo

**Affiliations:** 1 Rutgers Center for Tobacco Studies, New Brunswick, NJ 08091, USA; grafovib@sph.rutgers.edu (I.B.G.); delnevo@rutgers.edu (C.D.D.); 2 Department of Health Behavior, Society and Policy, Rutgers School of Public Health, Piscataway, NJ 08854, USA; 3 Rutgers Cancer Institute of New Jersey, New Brunswick, NJ 08091, USA

**Keywords:** price elasticity, public health policy, retailer scanner data, smokeless tobacco, moist snuff, taxation, tobacco, tobacco policy

## Abstract

Background: Little is known about how policies and industry activities impact smokeless tobacco demand. We examined how tobacco control policies and retail promotion may affect smokeless tobacco sales. Methods: We used Nielsen market-level retail scanner data for smokeless tobacco sales in convenience stores in 30 US regions from 2005 to 2010. Tobacco policy variables, including excise taxes, state tobacco control program expenditures, and clean indoor air laws, were merged to Nielsen markets. We estimated regression models for per capita unit sales. Results: Higher cigarette tax was significantly associated with lower sales volume of smokeless tobacco. Sales of smokeless tobacco in markets with a weight-based SLT excise tax were higher than in markets with an ad valorem tax. A higher average product price was associated with decreased sales overall but results varied by package quantity and brand. Conclusions: This study observed that smokeless tobacco products were both complements and substitutes to cigarettes. Thus, smokeless tobacco may act as complements for some population segments and substitutes for others. A weight-based tax generally favors premium smokeless tobacco products.

## 1. Introduction

Smokeless tobacco (SLT) in the US has traditionally been made in two dominant forms: chewing tobacco and moist snuff. Chewing tobacco is available in loose leaves, plugs, or twists of tobacco, and is placed between the cheek and gum or teeth while moist snuff tobacco (MST) is a finely ground tobacco packaged in cans or pouches, which can be sold dry (powdered form that is sniffed) or moist (placed between the lower lip or cheek and gum), and is sometimes used in teabag-like pouches. While chew was once more popular than MST, consumption of MST has exceeded chew since about 2000 and grown to make up more than 90% of the SLT market [1]. MST also leads all types of SLT in marketing expenditures [2]. Swedish-style snus, which is technically a MST product, is a more recent entry in the US SLT market and characterized as having a lower level of tobacco-specific nitrosamines than US MST products. These types of SLT as well as the different brands within each type vary widely in the amount of nicotine and nitrosamine [3,4,5]. SLT use increases the risk of multiple cancers, oral diseases, as well as cardiovascular disease [6,7,8]. The health risks associated with SLT are believed to be lower than the ones associated with conventional cigarettes [9] and experts estimate that low nitrosamine SLT products including snus are 90% less harmful than conventional cigarettes [10]. However, research has found that the nicotine levels of MST products increased over time for several brands making them potentially more dangerous. Despite the potential to adversely affect health, market share has grown for these brands [11]. A recent study estimated that the excess annual health care expenditures specifically attributable to SLT use among adults totaled $3.4 billion [12]. 

Policy interventions to reduce the harm from tobacco have primarily focused on cigarettes rather than other tobacco products like SLT and thus, the body of evidence, demonstrating the effectiveness of such interventions on use, including clean indoor air laws, taxation, and state-level comprehensive tobacco control programs, has largely been restricted to cigarettes. For example, increasing the price of cigarettes through taxation has been highly effective in reducing cigarette use both in frequency and prevalence [13]. Most studies of cigarette demand produce price elasticity estimates in the range of −0.3 to −0.5, implying that a 10% increase in price reduces overall consumption by about 3 to 5% with higher estimates for youth and young adults [14]. The limited evidence base for SLT products is consistent in showing that SLT demand is responsive to changes in SLT taxes and prices [15,16,17,18,19]. Older studies found mixed results on whether SLT use was a substitute or a complement to cigarette smoking while more recent work seems to more consistently point to SLT and cigarettes as complements, particularly among studies reporting sales elasticities [20,21].

In recent years, the price of tobacco products rose as a result of substantial increases in the state and federal tax rates of cigarettes and other tobacco products. On April 1, 2009, the federal tax rates on cigarettes and MST both increased by 158%, from 39 cents to $1.01 per pack of cigarettes and from 58.5 cents to $1.51 per pound of MST. Moreover, MST has traditionally been taxed in the US on an ad valorem basis (i.e., percentage of price) but in the last few years, numerous states changed the structure of MST taxation from ad valorem to weight-based. The SLT industry, in particular United States Smokeless Tobacco Company (USSTC) who is the dominant premium MST manufacturer, was a strong proponent of a weight-based MST tax in response to the shifting market share of SLT products after 2004 [22]. The threat that sub-premium, and in particular, deep discount brands, pose to premium brands’ (e.g., Skoal) market share prompted USSTC to engage in widespread lobbying efforts to change MST taxation to a weight-based system to reduce the price gap between premium and discount brands. While only two states taxed MST by weight prior to the year 2000 [22], as of July 1, 2014, 22 of the 49 states with a MST tax imposed a weight-based tax as well as the District of Columbia [23].

The limited research on the effect of indoor smoking restrictions on SLT use is also somewhat mixed. Ohsfeldt, et al. used data from the 1985 Current Population Survey (CPS) to examine the impact of state laws restricting smoking and found such laws had no effect on SLT use [16]. However, Mumford and colleagues, using CPS data from the 1990s, found a small impact from workplace smoking bans, finding decreased smoking among SLT users *and* SLT use among smokers [24]. More recently, Adams, Cotti, and Fhurmann examined the Behavioral Risk Factor Surveillance System (BRFSS) from 1995–2004 and their results suggested that SLT and cigarettes were substitutes, finding that a smoking ban in bars increased SLT use by 2.93% for smokers, nearly double the rate of SLT use among this group [25].

For state-level comprehensive tobacco control programs, which may also work to expand clean indoor air laws and increase tobacco prices through higher excise taxes, there is strong evidence that such expenditures are independently associated with decreases in cigarette smoking [26,27,28,29]. However, only one study explored the effect of state tobacco control expenditures on individual-level SLT use [30] using three different measures of per capita tobacco control program expenditures and they found that such expenditures were not associated with SLT use in young adults with the exception of one case in which current expenditures appeared to increase SLT use [30].

In addition to the expansion of tobacco control policies, there has been a fundamental shift in the SLT marketplace since many of these studies were conducted. In 2006, cigarette companies RJ Reynolds and Philip Morris as well as USSTC launched new SLT products to convert adult cigarette smokers to SLT use [31]. These new products were positioned as products to which smokers can switch, particularly those in states with smoke-free air laws. Indeed, USSTC’s investor materials indicate that the company viewed this as a growth opportunity, as “adult smokers are looking for an alternative [31].” The increasing popularity of pouch products (MST packaged in a teabag-like pouch, eliminating the need to spit) with existing users and a likely preference among smokers encouraged USSTC to re-launch its starter pouch product Skoal Bandits in 2006. In 2009, pouch product line extensions were also introduced by popular discount brands (e.g., Grizzly, Longhorn). Overall, sales of portion pouch MST products increased by 333.8% between 2005 and 2011 and represented 14.5% of the MST market share in 2011 [32]. In addition, flavors have contributed to the growth in MST. Sales of flavored MST products increased by 72.1% between 2005 and 2011 and contributed to approximately 60% of the growth in the MST category [32]. The MST category, traditionally dominated by two major premium brands, Skoal and Copenhagen, has more recently been led by value/discount brands such as Grizzly, which went from the third top selling brand in 2005 (with a 14.8% market share) to the number one selling brand in 2009, moving ahead of the premium brand market leaders, Copenhagen and Skoal [32].

The SLT industry in the US has transformed the way that it markets and manufactures MST products. In addition, research on the impact of traditional tobacco control activities is scant and most studies were conducted prior to the implementation of many policy changes including the growth of clean indoor air laws, increased taxation, and the shift away from ad valorem MST excise taxes to a weight-based MST tax structure. The objective of this analysis was to link policy variables such as tax, price, state tobacco control funding, and clean indoor laws to market-level data on MST sales to evaluate the impact of these policies on aggregate MST sales between 2005 and 2010. This work builds on more recent literature using retail scanner data to study consumer demand for non-cigarette tobacco products [20,21] and makes several important additions by incorporating specific policy and product characteristics. 

## 2. Materials and Methods

**Figure 1:**
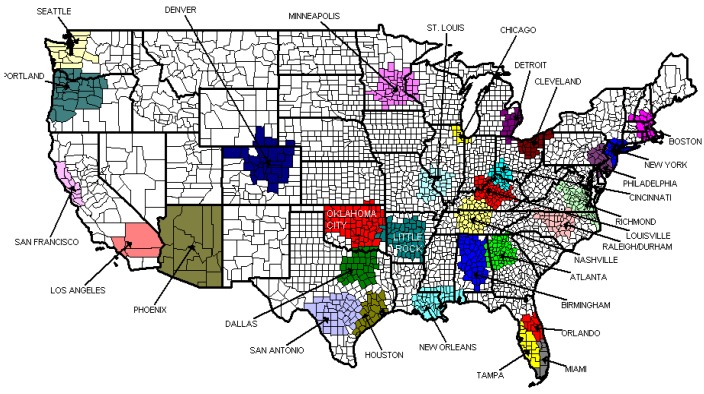
Nielsen’s 2005–2010 Convenience Track market areas.

The primary data source for the outcome variable of MST sales was biannual market scanner data from 2005–2010 Nielsen's Convenience Track system, which tracks sales data from a sample of convenience stores that are representative of all convenience store types. Using a proprietary mechanism, Nielsen applies sample weights to scanned retailer data before reporting. This analysis includes MST sales in US convenience stores from 2005 to 2010 for 30 market areas, totaling 360 unique biannual market observations. The market areas are defined by Nielsen and are collections of counties that usually contain at least one large metropolitan area (see Figure 1). Based on Nielsen data from 2008, the average number of counties in a market area is 32 (range 4–62). Market areas contain an average 5.9 million people (range 1.97 million–20.1 million) and collectively cover approximately 58% of the U.S. population. 

However, the market areas do not generally conform to convenient geographic areas and often cross state borders. Nine markets are contained entirely within a single state and 21 markets include at least one county from more than one state. By linking Census Bureau county-level population data to the counties that made up each Nielsen market area, we derived the proportion of the total market population that resides in each state(s) that contribute to the market and weighted the policy variables accordingly to estimate exposure to specific tobacco policies at the market level (described below in measures). 

### 2.1. Measures

#### 2.1.1. Moist Snuff (MST) Sales 

The dependent or outcome variable in these analyses were product-level per capita sales volume constructed by dividing the total MST sales volume in a market by the market population size for each year. The population size for each Nielsen market was calculated by linking Census Bureau county-level population data for each year (2005–2010) to the counties that comprised each Nielsen market area. MST unit sales were also multiplied by the total ounces reported for the product package. All dollar variables were inflation adjusted using the Consumer Price Index (CPI) for 2010 as reported by the Bureau of Labor Statistics [33]. 

#### 2.1.2. Tobacco Industry Practices

We included tobacco product attributes or characteristics in the analysis including brand, flavoring, form (in this case, pouch), and packaging (e.g., pack size). In terms of retail promotions, Nielsen data include promotion codes for bonus ounces, bonus unit, cents off, gift, and pre-priced; promoted MST products were coded as yes (or a value of 1) if designated as sold under any one of the aforementioned promotion codes. 

#### 2.1.3. Tobacco Control Policies

To examine the potential influence of tobacco control policies on the demand for MST across markets, the analyses included state-level contextual data for the time period of interest (2005–2010) compiled from several sources. State level cigarette excise tax and MST excise taxes (both the size or value of the MST tax as well as the type of MST tax) for each year were obtained from CDC’s State Tobacco Activities Tracking and Evaluation (STATE) System. If a Nielsen market consisted exclusively of counties in states that taxed MST on an ad valorem basis, the market was coded as ad valorem MST tax market only in that year. If a Nielsen market consisted exclusively of counties in states that taxed MST based on weight, the market was coded as a weight-based MST tax market only; and finally, if the Nielsen market contained counties in states that taxed on an ad valorem basis and by weight, the market was coded as a mixed MST tax market.

Average MST price for each product came from the Nielsen market scanner data and reflects excise tax paid by the consumers and any retail promotion or discount applied at the point of sale but does not include sales tax. 

Annual state tobacco control program expenditures was obtained from a published analysis of state funding for tobacco control and expenditure data was adjusted to a fiscal year ending June 30 [34]. Per capita tobacco control program expenditures were derived by dividing these expenditures by the Nielsen market population size for each year.

Following previous approaches [28,29,35], clean indoor air policy was measured as the annual percentage of the state population covered by state or local smoke-free air laws that banned smoking in all workplaces, restaurants, or bars. These data were provided by the American Non-Smokers’ Rights Foundation U.S. Tobacco Control Laws Database^©^.

### 2.2. Linking Policy Level Variables to Market Data 

State-level tobacco policy variables were matched to Nielsen market areas by state and time. For markets that include more than one state, a weighted average of these regulatory measures was calculated using the proportion of the market population residing in each state. To derive the proportions of the market population residing in each state, we used county population estimates from 2005 to 2010 for the counties that made up each Nielsen market. For example, the New York market includes portions of New York as well as New Jersey and Connecticut. Thus, for the New York Nielsen market area, state-level policy variables for the state of New York were weighted by the state’s proportion of the market area—about 63% of this Nielsen market area is in the state of New York. Similarly, state-level policy variables for New Jersey and Connecticut were weighted by the states’ proportion of the New York Nielsen market area—about 32% and 5%, respectively. These weighted averages more appropriately reflect the potential impact of a particular state-level tobacco control policy on MST sales in a particular Nielsen market area. 

### 2.3. Sample Size

We imposed several restrictions on the pooled cross-sectional sample. We used Nielsen sales data in six-month periods from January 2005 through December 2010. The initial sample size included 71424 SLT UPC sale records (i.e., sales data for each UPC product code for each six months period between 2005 and 2010 for each market). Given that MST made up over 90% of the SLT market during this time period, the sample was then restricted to MST products only (including snus) which reduced the sample to 60845 MST UPC sale records. For some model estimations, the sample was stratified by brand and/or pack size (i.e., single, 2 pack, 5 pack) and thus, sample sizes varied between 4104 and 49288 records. 

### 2.4. Model Specifications 

To examine the relationship between MST sales and retail promotion and tobacco control policies, we estimated the following ordinary least squares (OLS) regression:

*MSTsales_its_* = *α_0_* + *α_1_Spending_it_* + *α_2_V_it_* + *α_3_CIA_it_* + *α_4_Promotion_it_* + *α_5_Time_t_* + *α_6_Season_it_* + *u_it_*

where MST pre capita sales *MSTsales_its_* in market *i* in the time period *t* for season *s* depend on per capita tobacco control program expenditures (*Spending_its_*) in the state where market *i* was located; state-level policies related to tobacco which affect price (*V_its_*) where V was a vector of such elements; clean indoor air (*CIA_its_*); MST retail promotions ascertained from Nielsen data (*Promotion_its_*); year effects (*Time_ts_*), seasonal effects (*Season_it_*) and an error term (*u_its_*). Seasonal effects (*season*) reflected whether sales occurred in the first (January to June) or second half (July to December) of year *t.*

The outcome variable of MST sales was estimated in units (adjusted by package size or total ounces). The price vector *V_its_* included the MST excise tax cigarette excise tax, and average MST product price. We used several model specifications to test the use of alternative variables for average MST price (median price, unit price, etc). Because of the high correlation between MST excise tax and MST price, the variable reflecting the *size* (or the value) of MST excise tax was removed from the final model to minimize multicollinearity. The variable depicting the type of MST excise tax in the market (e.g., ad valorem only, weight-based tax only, or mixed) was retained in the final model. 

### 2.5. Alternative Model Specifications 

Given the role that package quantity may play on consumer behavior, we also re-estimated the models described above by pack size (i.e., single, two, and five packs) and by product type (premium and non-premium MST products), and by Census region (Northeast, Midwest, South, and West). We also repeated the analysis above separately for the top three selling MST products (Skoal, Copenhagen, and Grizzly). 

## 3. Results

### Market Characteristics 

The *size* of state-level cigarette and MST excise taxes varied within and across Nielsen markets. During the time period of interest, state level MST excise taxes also changed in structure and thus, the *type* of excise tax also varied within and across markets. In 2005, a total of 19 Nielsen markets consisted exclusively of states which taxed MST on an ad valorem basis only, representing nearly 60% of the market population compared to 13 such markets in 2010, reflecting 42% of the market population. Markets consisting exclusively of states that taxed MST based on weight increased to 7 markets in 2010 or 22.8% of the market population) from 2 in 2005 (or just 5.2% of the market population). The number of markets that included both types of MST excise tax structures remained relatively consistent across years. 

Table 1 reflects the tobacco control policy characteristics of the combined Nielsen markets between 2005 and 2010. In markets where smokeless products were taxed on an ad valorem basis (% of price), the mean value of MST excise tax rates remained relatively stable, at 27.18% over the time period of interest (range: 0–75%). In markets where MST products were taxed based on weight (cents), MST excise tax rates increased in mean value incrementally from 2005 to 2009 (from 1 to 10 cents) but then increased considerably in 2010 (27 cents per ounce). The proportion of the population in an average market that was covered by comprehensive clean indoor air policies grew substantially, from nearly 10% in 2005 to almost 40% of the population in 2010. The mean value of the cigarette excise tax in these markets also increased from 2005 to 2010, from $0.90 cents to $1.19. However, there was also considerable variation in the cigarette excise tax across markets (range = $0.23 to $3.76). Per capita tobacco control program expenditures fluctuated somewhat during this period, with a mean value of $2.21 dollars across the time period, with the lowest level of funding in 2010 (range = $0.11 to $7.21. 

Table 2 reports the overall MST sales in the combined Nielsen markets between 2005 and 2010 as well as other market characteristics for MST. Total unit and dollar sales of MST increased over this time period while both the average and weighted average unit prices declined over time. Per capita unit consumption increased by about 44% while per capita consumption measured in dollars increased by nearly 36% over this time period. In addition, the percent of MST sales occurring under a retail promotion and those that were multipack decreased, although in both cases made up a small percent of the overall market. Pouches and non-premium brands, however, increased as a percent of overall MST unit sales over this time period. 

Table 3 shows that markets with a higher percent of a market population covered by smoke-free air policies was significantly associated with lower per capita MST unit sales. In addition, a higher cigarette tax was significantly associated with lower per capita unit sales of MST. Per capita state level tobacco control program expenditures had a positive and significant impact on per capita MST unit sales; that is, higher tobacco control expenditures were associated with increased MST unit sales. Thus, higher spending on tobacco control was likely to decrease cigarette smoking but increased MST sales. Results indicated that a higher average product price for MST was associated with lower MST unit sales. However, the stratified analysis showed that this relationship was not uniform across single and multi-pack products. A higher average price for five-pack MST products also resulted in lower unit sales. In contrast, a higher average price was related to higher unit sales among single pack and two-pack products. It appears that a negative relationship between MST price and sales volume is primarily driven by larger product quantities of MST. If the price for five pack MST products increases, the quantity demanded decreases, which could suggest that consumers may not have adequate substitutes for their preferred product. However, the vast majority of MST products were sold in single packs, ranging from 96% to 99% of the market depending on year. Therefore, the relationship between MST price and sales volume is positive for the overwhelming proportion of the MST market.

The results in Table 3 show that sales volume was higher in markets with mixed and weight based-only tax structure than in markets with ad valorem tax structure. This relationship was statistically significant for the pooled sample as well as the single and two pack product subsamples. This relationship was also positive in the five pack product subsample but the coefficient was small and not statistically significant. This may suggest that large volume consumers were simply less sensitive to differences in the MST tax structure. Sales volume of products sold with the retail promotion, which implies a price discount (most frequently cents-off), was significantly lower than sales volume of products sold without retail promotion, except for five-pack MST products where the relationship was negative but not statistically significant. Thus in most cases, the increased likelihood of a retail promotion resulted in decreased per capita MST unit sales. For per capita MST unit sales overall, there were no significant differences by year. 

Own price elasticities for MST and cross-tax elasticities with respect to cigarette tax are shown in Table 4. The own price elasticity of demand for moist snuff overall and five pack products was negative and ranged from −0.155 to −1.258. The own price elasticity of demand for MST single and two pack products was positive and ranged from 0.582 to 1.510. The cross-tax elasticities, positive for substitutes and negative for complements, measure the change in the demand for MST with respect to a change in the cigarette tax. In all cases, the demand for MST products decreased in response to higher cigarette tax. Overall, the demand for MST declined in response to a higher cigarette tax (cross tax elasticity [with respect to cigarette tax] = −0.294 for MST unit sales). That is, a 10% increase in the cigarette tax resulted in a nearly 3% decline in MST demand as measured in unit sales. Similarly for single, two, and five pack products, MST unit sales declined in response to a higher cigarette tax; that is, a 10% increase in the cigarette tax resulted in declines ranging from 2.8% to 3.97% reductions in MST unit sales. 

Table 5 presents the model examining the relationship between tobacco control policies and retail promotions and per capita MST sales volume of single packs of premium and non-premium brands overall as well as the top selling brands of Copenhagen, Skoal, and Grizzly. As shown, there appeared to be a pattern in that the coefficients for smoke-free air policies, cigarette tax, type of MST tax, and retail promotion were nearly all greater in size for premium brand relative to non-premium brand single pack MST products. Among premium brands, smoke-free air policies were significantly negatively associated with per capita single pack MST unit sales. Higher cigarette tax also had a negative and significant impact on per capita unit sales across all single pack MST products, both premium and non-premium brands. The impact of clean indoor air policies and cigarette taxes on MST unit sales indicate complementarity between MST and cigarettes while the results of the impact of state-level tobacco control expenditures on overall MST unit sales indicate substitutability. This suggests that among single pack MST products the positive effect of tobacco control expenditures on MST sales volume was primarily driven by non-premium brands rather than premium brands. Consistent with differences observed between premium and non-premium brands in relation to other tobacco policies, a positive relationship between MST price and unit sales was driven primarily by premium brands. The relationship between MST price and sales volume was not statistically significant for non-premium brand MST products and even negative for Grizzly brand. So in addition to differences in consumers of varying MST package quantities, it appears that consumers of premium and non-premium products are also different.

Overall, a mixed tax market resulted in significantly higher per capita single pack unit sales across all brands relative to markets with only an ad valorem tax. There was a statistically significant and positive association between a weight-based only tax market and most brands, although the strength of the association was strongest for Copenhagen products. Retail promotions were negatively related to per capita unit sales of premium and non-premium products as well as the three leading brands. Thus, the impact of tobacco control policies varies across brands and Grizzly, though priced as a non-premium/value brand, often responds like a premium brand in the marketplace. Given the geographic differences in use of MST, we also re-estimated the model from for single pack unit sales by the four Census regions—Northeast, Midwest, South, and West. Although previously negative and statistically significant, the impact of smoke-free air policies on MST unit sales weakened and lost significance in the South while maintaining a negative and significant association on MST unit sales in the other regions of the U.S (data not shown). The direction of the relationship between MST unit sales and cigarette tax changed in the Northeast and West regions (from negative to positive but not statistically significant) which would indicate that MST and cigarettes may be substitutes rather than complements like in the Midwest and South. The results are perhaps consistent with the overall climate for tobacco control policies in the Southeast in that states in this region have been slower to adopt comprehensive clean indoor laws or high cigarette excise taxes. The direction of the relationship also changed, from positive to negative, for MST unit sales and per capita tobacco control expenditures in the Northeast and lost statistical significance in the Midwest.

## 4. Discussion

Despite many public health efforts to discourage tobacco use during the time period of interest, the tobacco industry continued to effectively market SLT products resulting in increased MST sales. Between 2005 and 2010, many local, state, and federal-level tobacco control policies were enacted including expansion of clean indoor air laws, higher excise taxes for combustible and SLT products, and investment in tobacco control programming. The percentage of the population in Nielsen market areas covered by state-level comprehensive clean indoor air laws grew from less than 10% to over 40% of the population and the mean state-level cigarette excise tax in these areas was raised from $0.90 to $1.19, both representing a 30% increase. However, the average and weighted unit prices for MST in these market areas actually declined over this period while total unit and dollar sales and per capita consumption of MST increased substantially. In addition, the percent of MST unit sales that were non-premium brands or sold in pouches grew over this time period. 

In addition, policy and regulation at the federal level have increased restrictions on the sales, marketing, distribution, and taxation of cigarettes. Specifically, under two tobacco control laws that became effective in 2009 - the Children's Health Insurance Program Reauthorization Act (CHIPRA) and the Family Smoking Prevention and Tobacco Control Act (Tobacco Control Act) - cigarettes are subject to stricter tax and other regulations than some other tobacco products. For example, the Tobacco Control Act prohibited characterizing flavors in cigarettes but these flavors are still available in SLT products. Much larger increases in federal excise tax rates were implemented by CHIPRA on cigarettes in comparison to MST. In addition, many states shifted from an ad valorem MST excise tax to a weight-based system which in some cases reduced the tax burden for MST products. 

A question posed by this analysis was what was the effect of retail promotion and tobacco control policies on MST sales across various markets? Lower demand for MST was anticipated in markets with high MST price while growth in MST demand was expected in markets with high cigarette taxes, higher levels of expenditures for tobacco control programming, broader protection of clean indoor air, and more retail promotions. Overall, a higher average MST price was associated with a decline in MST sales but there were differences by package quantity and brand. Single and two pack MST product unit sales actually increased while five pack products decreased in response to a higher average MST price. For premium brands, however, a higher MST price was associated with increased MST sales. The type of tax structure was also significantly associated with MST sales volume in that markets with a mixed or weight-based only MST excise tax significantly increased MST sales relative to a market with an ad valorem MST excise tax only. There were larger effect sizes for premium brand MST sales volume and revenue in markets with a weight-based MST excise tax only.

A higher cigarette tax was significantly associated with lower per capita MST unit sales, suggesting complementarity between MST and cigarettes. Previously reported cross-price elasticities for SLT and cigarettes were −0.045 [20], −0.0434 (although not significant) [21], and −0.77 [36]. The cross-tax elasticities for cigarettes and MST found in this study were lower (−0.22 for unit sales) but also varied by package quantity. MST has generally found to be less elastic relative to combustible products [20,21]. In terms of other policy interventions, both complimentary and substitution were observed, similar to previous literature that found varying consumers’ responses to tobacco control policies. A higher percent of a market population covered by smoke-free air policies was, in most cases, associated with lower per capita MST sales which suggests complementarity. Overall, higher tobacco control expenditures were associated with increased MST sales, which suggests substitution, although such expenditures had no impact on premium MST brands. In addition, it appeared that any relationship to year was occurring in the first few years of the study period (before 2009). The results also indicate that there are brand differences in that the effect sizes found for premium brands were larger relative to non-premium brands for many tobacco policy interventions including the percent of population covered by smoke-free air policies, cigarette tax, the type of MST tax structure, and retail promotion.

Could tobacco control spending nudge cigarette smokers to non-premium brands of smoke-free products? Tobacco control programs have undoubtedly focused on reducing cigarette smoking with few efforts targeting non-cigarette tobacco products. The only other study to specifically examine the impact of per capita state-level tobacco control program expenditures did so on individual SLT use among college students and found that current spending was associated with increased SLT use but two other measures of spending (lagged, and sum of current and lagged) revealed no relationship [30]. Previous research has recognized that new SLT products were developed to target cigarette smokers to promote switching or dual use [37]. In a recent analysis of two longitudinal cohorts of the TUS-CPS, male recent former smokers in the 2010–2011 cohort were more likely to become SLT users than those in the 2002–2003 cohort but overall, smokers were unlikely to switch to other forms of tobacco compared to SLT users [38]. Tobacco control programs encompass a variety of activities and interventions and so it is difficult to pinpoint why program expenditures may have a differential impact on MST products. 

Not surprisingly, markets with a mixed MST excise tax structure (which would include weight-based MST taxes) or a weight-based only tax structure favored premium brands to a greater degree than other MST brands. A weight-based only MST excise tax, which is essentially a tax on quantity or a per unit tax, ignores the price of the product and reduces the tax on premium (i.e., higher priced) products whereas a tax based on the percentage of the sale price imposes a higher tax on premium products for obvious reasons. The growing market share of discount MST brands was often acknowledged by tobacco industry representatives arguing for weight-based taxation. In particular, U.S. Smokeless Tobacco Company (UST), the manufacturer of two leading premium MST brands, Copenhagen and Skoal, strongly advocated that states move to a weight-based MST tax in order to compete more effectively with low-priced brands [39].

Despite the advantage for premium brands under a weight-based MST tax structure, the value brand of Grizzly has challenged popular premium brands for market share including, the well-established market leaders of Copenhagen and Skoal, both premium brands. These data suggest that in some ways Grizzly responds more like a premium brand. For example, state spending on tobacco control initiatives had no effect on the sales volume of premium brand MST products or Grizzly while it had a positive effect on non-premium brands overall. Previous research suggests that Grizzly’s popularity, particularly among youth, may be due to low price, advertising and added flavorings as well as faster nicotine dependence because of high nicotine content [11]. 

In line with the work of Zheng, et al., additional analysis by U.S census region found differential impact on MST demand [20]. Zheng, et al. found the South to be the most elastic region for SLT [20]. Interestingly, this study found that cigarette taxes and clean indoor air laws did not significantly change demand for MST in South. Also, markets with weight-based MST tax only did not exist in the Northeast and Midwest regions but MST unit sales in the South and West regions increased under a weight-based MST tax system. While not precise, the results give us some indication that there are cultural and demographic differences that can influence the success or effectiveness of tobacco policy interventions. 

This study examined the effect of retail promotion and tobacco control policies on MST sales. Most studies on the impact of tobacco control policies focus on cigarettes, so the research presented here fills important gaps in the tobacco control literature. Perhaps most importantly, the series of analyses incorporated specific brands and package size into the nature of consumer substitution between tobacco products. Such measures may be needed to more accurately describe the demand for MST, and potentially other non-cigarette tobacco products, more fully. 

## 5. Limitations

Despite the importance and unique contribution of this work, it is not without methodological limitations. The market-level scanner data for MST sales was limited to convenience stores. While the vast majority of MST products are sold in convenience stores, evidence suggests that the elasticity of demand is different according to the outlet in which it is sold [21,40]. Also, convenience store market areas may include several states in one market area and do not represent any one state; thus, while we attempted to estimate tobacco policy exposure at the market level, the study outcomes were based on market areas and not entire states, the level at which many tobacco policies are implemented. In addition, retailer scanner data may be more elastic given that sales elasticities may be more responsive to price than consumption elasticities (e.g., stockpiling for price discounts). In addition, this is analysis did not include data on advertising expenditures which previous research shows can influence demand for tobacco products. For example, Zheng et al. (2017) found that elasticity of SLT magazine advertising on its own demand was 0.002 while Dave & Saffer (2013) reported that magazine advertising elasticity on SLT demand was 0.06 [20,36]. In addition, Zheng et al. (2017) also found that such advertising increased SLT demand in the Midwest and North but not the South or West regions of the U.S [20]. Finally, because the data reflect sales in aggregate, we could not examine differences by important individual characteristics such as gender, race, ethnicity, age, education and income.

## 6. Conclusions 

The evidence base leans more heavily toward MST and cigarettes acting as complements (e.g., as the price of cigarettes increase, the demand for MST increases). However, we observed that MST can act as both compliments and substitutes to cigarettes. And it appears that non-premium brand MST products are more likely to exhibit the properties of substitution relative to premium brand MST products. A better understanding of the relationship between cigarettes and non-cigarette tobacco products has important policy implications. For the most part, policy interventions aimed at reducing cigarette smoking such as cigarette taxation and clean indoor air policies appear to decrease MST sales and so should work effectively to reduce the use of MST products. However, results may vary based on specific program components as well as by consumer segments and region. 

There is substantial product differentiation by package quantity, presence of flavors, and branding in the tobacco marketplace. These results provide further evidence that tobacco companies target consumers in rather sophisticated ways and different products are aimed at different segments of the consumer market. Tobacco companies appear to be well aware of differences in the population of consumers for these products and target them accordingly. For example, sales of five pack MST products decreased when MST price increased while sales of single pack MST products increased. 

Policy restrictions could target any further attempts to reinforce quality differences between various tobacco brands and consumer segments. For instance, pricing or the display of pricing at the point of sale give prominence to particular brands and can be effective promotional strategies. Limiting the display of price at retail or standardizing the unit price may also limit perceived differences between consumer market segments. State-level tobacco policies can have an immediate impact on leveling the playing field for all MST products with regard to taxation, particularly considering that tax rates vary both in size and structure at the state level. Some recommend setting a minimum tax on MST products equivalent to the per pack tax on cigarettes along with a high ad valorem tax rate when possible as an effective way to increase the price of these products [41,42]. 

One of the important policy implications here is the recognition that blanket recommendations for tobacco policy may not be as effective as a tailored policy approach. Tobacco companies are clearly using tailored marketing strategies to target different consumer segments. Population-based tobacco control efforts, such as policy change, are effective, have made an enormous difference in tobacco consumption and must be continued, but should also be designed to influence behavior change for maximum impact. The variation in consumer response to tobacco policy and marketing variables is in stark contrast to the blanket approaches often used by tobacco control policymakers. In the context of an increasing diverse tobacco marketplace, policies and regulations that target a single product or consumer segment may have various benefits and consequences for the use of different MST products (e.g., brand, size, etc.) as well as use of *other* tobacco products dependent on within and cross product substitutions. 

The use of two or more tobacco products has increased over time, especially among young adults [43,44,45,46]. A recent estimate suggests that approximately 40% of tobacco users in the U.S, both adults and youth, used at least two types of products [47]. Dynamic complementarity may explain why users of multiple tobacco products tend to use tobacco products more frequently and exhibit greater tobacco dependence than single tobacco product users [48]. Federal, state, and local policy makers must consider how policy action may affect the use of other tobacco products by altering cross substitution.

## Figures and Tables

**Table 1:** Policy characteristics of 30 Nielsen C-track markets, 2005–2010.

Policy Measure	2005		2006		2007		2008		2009		2010	
	Mean	sd	Mean	sd	Mean	sd	Mean	sd	Mean	sd	Mean	sd
MST excise tax, ad valorem (% of price) ^a^	27.84	21.32	27.52	20.96	26.99	21.07	26.56	20.88	28.84	26.62	25.81	28.81
MST excise tax, weight (cents) ^b^	$0.01	$0.02	$0.02	$0.05	$0.03	$0.07	$0.04	$0.10	$0.10	$0.28	$0.27	$0.55
Pop. covered by 100% smoke-free air laws (%)	9.8%	22.0%	17.9%	30.8%	27.9%	35.6%	32.3%	37.2%	35.1%	38.4%	39.3%	40.1%
Cigarette excise tax (dollars)	$0.90	$0.58	$0.88	$0.58	$0.99	$0.58	$0.98	$0.61	$1.10	$0.61	$1.19	$0.70
Per capita TC program expenditures (dollars) ^c^	2.21	1.86	2.27	2.00	2.12	1.79	2.47	1.61	2.25	1.67	1.98	1.60

MST = Moist Snuff; TC= tobacco control. Note: Values reflect the mean across all product-market observations; dollars indexed to 2010 to adjust for inflation; ^a^ Averages calculated only for markets with an ad valorem MST excise tax; ^b^ Averages calculated only for markets with a weight-based MST excise tax; ^c^ Per population capita calculated at the market population level.

**Table 2:** MST sales and product characteristics in 30 Nielsen C-track markets, 2005–2010.

Sales or Product Characteristic	2005	2006	2007	2008	2009	2010	% Change
Total MST sales, units	280,267,499	313,391,993	344,145,225	360,057,323	382,279,066	424,687,624	51.5%
Total MST sales, dollars	$1,236,287,214	$1,315,555,514	$1,387,192,985	$1,376,272,342	$1,402,009,310	$1,573,517,135	27.3%
Average MST unit price	$3.95	$3.88	$3.83	$3.77	$3.61	$3.71	−6.1%
Average weighted MST unit price ^a^	$4.41	$4.20	$4.03	$3.82	$3.67	$3.71	−15.9%
Per capita MST consumption, units ^b^	1.65	1.82	1.98	2.05	2.16	2.38	44.2%
Per capita MST consumption, dollars ^b^	$6.50	$7.08	$7.60	$7.76	$7.80	$8.82	35.7%
% of MST unit sales with a retail promotion	2.2%	2.1%	1.9%	2.8%	2.1%	2.2%	0.0%
% of MST unit sales that were multipack	1.5%	2.5%	3.5%	3.9%	1.3%	0.7%	−53.3%
% of MST unit sales that were 1.2 oz	88.8%	88.4%	88.3%	87.8%	86.4%	85.1%	−4.2%
% of MST unit sales that were pouches	5.4%	6.0%	6.9%	7.9%	10.4%	13.1%	142.6%
% of MST unit sales that were premium brand	68.3%	63.1%	59.7%	57.0%	56.0%	57.6%	−15.7%

MST = Moist Snuff. Note: Averages reflect the mean across all product-market observations; dollars indexed to 2010 to adjust for inflation; ^a^ Weighted price by product market share; ^b^ Per population capita calculated at the market population level.

**Table 3:** Effect of tobacco control policies & retail promotions on per capita MST sales (Units) overall and by pack size, 2005–2010.

Policy Measure	Overall	Single Pack	2 Pack	5 Pack
Smoke-free air policy	−0.00276 ^***^	−0.00344 ^***^	−0.00208 ^***^	−0.000114 ^***^
	(0.000378)	(0.000466)	(0.000314)	(0.0000300)
Cigarette tax	−0.00218 ^***^	−0.00354 ^***^	−0.000715 ^***^	−0.0000469
	(0.000220)	(0.000269)	(0.000182)	(0.0000202)
Per capita state TC spending	0.000234 ^***^	0.000195	0.000110	0.0000336 ^***^
	(0.0000625)	(0.0000768)	(0.0000447)	(0.00000560)
Average MST price	−0.000310 ^***^	0.00161 ^***^	0.000458 ^***^	−0.0000137 ^***^
	(0.0000152)	(0.0000796)	(0.0000437)	(0.00000143)
Ad valorem MST tax only (referent)				
Mixed MST tax	0.00147 ^***^	0.00294 ^***^	0.000959 ^***^	0.0000110
	(0.000235)	(0.000289)	(0.000189)	(0.0000207)
Weight-based MST tax only	0.00381 ^***^	0.00522 ^***^	0.00221 ^***^	0.0000599
	(0.000373)	(0.000450)	(0.000292)	(0.0000361)
Retail promotion	−0.00910^***^	−0.00996 ^***^	−0.00109 ^***^	−0.000171
	(0.000271)	(0.000314)	(0.000208)	(0.0000892)
Constant	0.0124 ^***^	0.00716 ^***^	−0.000810	0.000283 ^***^
	(0.000396)	(0.000542)	(0.000387)	(0.0000521)
r2	0.0297	0.0385	0.0965	0.0321
*N*	60,845	49,288	4104	6507

Model controls for time; Standard errors in parentheses; MST = Moist Snuff; TC = tobacco control; ^***^ *p* < 0.001.

**Table 4:** Estimated elasticities of moist snuff and cigarettes.

MST Unit Sales	*N*	MST own Price Elasticity	Cross-Tax Elasticity (Cigarette Taxes)
Total	60,845	−0.222	−0.294
Single pack	49,288	0.582	−0.397
2 pack	4104	1.038	−0.283
5 pack	6507	−1.258	−0.281

MST = Moist snuff.

**Table 5:** Effect of tobacco control policies & retail promotions on per capita MST sales (Units) of single packs: results for brands, 2005–2010.

Policy Measure	Non-Premium	Premium	Skoal	Copenhagen	Grizzly
Smoke-free air policy	−0.00295 ^***^	−0.00445 ^***^	−0.00381 ^***^	−0.0164 ^***^	−0.000147
	(0.000549)	(0.000815)	(0.000580)	(0.00356)	(0.00297)
Cigarette tax	−0.00272 ^***^	−0.00443 ^***^	−0.00249 ^***^	−0.0117 ^***^	−0.00888 ^***^
	(0.000318)	(0.000473)	(0.000334)	(0.00210)	(0.00188)
Per capita TC spending	0.000440 ^***^	−0.000131	−0.0000939	0.000164	0.000560
	(0.0000898)	(0.000137)	(0.0000929)	(0.000582)	(0.000555)
Average MST price	−0.0000736	0.00222 ^***^	0.000458 ^***^	0.00854 ^***^	−0.00425
	(0.000108)	(0.000206)	(0.000152)	(0.00124)	(0.00185)
Ad valorem tax only (referent)					
Mixed MST tax	0.00213 ^***^	0.00407 ^***^	0.00357 ^***^	0.00867 ^***^	0.0108 ^***^
	(0.000339)	(0.000516)	(0.000358)	(0.00231)	(0.00201)
Weight-based MST tax only	0.00291 ^***^	0.00862 ^***^	0.00635 ^***^	0.0235 ^***^	0.00580
	(0.000532)	(0.000791)	(0.000562)	(0.00340)	(0.00294)
Retail promotion	−0.00734 ^***^	−0.0140 ^***^	−0.0120 ^***^	−0.0291 ^***^	−0.0315 ^***^
	(0.000397)	(0.000533)	(0.000370)	(0.00240)	(0.00214)
Constant	0.00816 ^***^	0.00978 ^***^	0.0112 ^***^	0.00866	0.0467 ^***^
	(0.000619)	(0.00130)	(0.000915)	(0.00706)	(0.00544)
r2	0.0201	0.0661	0.123	0.101	0.0922
*N*	29,592	19,696	11,289	3779	4363

Model controls for time; Standard errors in parentheses; MST = Moist Snuff; TC = tobacco control; ^***^ *p* < 0.001.
